# Overexpression of mouse *TTF-2* gene causes cleft palate

**DOI:** 10.1111/j.1582-4934.2012.01546.x

**Published:** 2012-09-26

**Authors:** Tian Meng, Jia-Yu Shi, Min Wu, Yan Wang, Ling Li, Yan Liu, Qian Zheng, Lei Huang, Bing Shi

**Affiliations:** a State Key Laboratory of Oral Diseases, West China School of Stomatology, Sichuan UniversityChengdu, China; b Department of Cleft Lip and Palate Surgery, West China School of Stomatology of Sichuan UniversityChengdu, China; c Department of Biochemistry and Molecular Biology, University of North Dakota School of Medicine and Health SciencesGrand Forks, ND, USA; d Laboratory Animal Center of Sichuan UniversityChengdu, 610041, China; e Department of Oral and Maxillofacial Surgery, Guangzhou First Municipal People's HospitalGuangzhou, China

**Keywords:** TTF-2, overexpression, transgenic mouse, cleft palate

## Abstract

In humans, mutations of the gene encoding for thyroid transcription factor-2 (TTF-2 or FOXE1) result in Bamforth syndrome. Bamforth syndrome is characterized by agenesis, cleft palate, spiky hair and choanal atresia. *TTF-2* null mice (*TTF-2^−/−^*) also exhibit cleft palate, suggesting its involvement in the palatogenesis. However, the molecular pathology and genetic regulation by TTF2 remain largely unknown. In the present study, the recombinant expression vector pBROAD3-*TTF-2* containing the promoter of the mouse *ROSA26* gene was created to form the structural gene of mouse *TTF-2* and was microinjected into the male pronuclei of fertilized ova. Sequence analysis confirmed that the *TTF-2* transgenic mouse model was established successfully. The transgenic mice displayed a phenotype of cleft palate. In addition, we found that TTF-2 was highly expressed in the medial edge epithelium (MEE) from the embryonic day 12.5 (E12.5) to E14.5 in *TTF-2* transgenic mice. These observations suggest that overexpression of TTF-2 during palatogenesis may contribute to formation of cleft palate.

## Introduction

The formation of the secondary palate (palatogenesis) in mammals involves the orchestration of several processes to produce the correct separation of the oral and nasal cavities. Failure of palatogenesis results in cleft palate, one of the most common birth defects in humans [1].

Murine palatogenesis takes place between embryonic days 12.5 and 15.5 (E12.5-E15.5) [1]. Palatal shelves grow out bilaterally from maxillary prominences. Around E14.5, palatal shelves rapidly elevate to a horizontal position, and become adherent in the midline, before opposing palatal shelves finally fuse. During the initial stage of the fusion process, the epithelia covering the tip of each palatal shelf (MEE) adhere to form a midline epithelial seam (MES) [1]. The MES disappear through the combination of programmed cell death [2–4], epithelial-mesenchymal transformation (EMT) [5–7] and migration to the oral and nasal palatal epithelia [8]. Failure of any of these processes can result in isolated cleft palate.

Cleft palate can be caused by mutations in a variety of genes including transcription factors, growth factor receptors, extracellular matrix components, and cell surface adhesion molecules [9–17] and decreased malonylcarnitine [18]. Thyroid transcription factor-2 was first identified as a thyroid-specific DNA binding factor recognizing thyroglobulin (Tg) [19] and thyroperoxidase (TPO) [20] promoters. Zannini *et al*. [21] cloned the rat cDNA encoding TTF-2 and demonstrated that TTF-2 is a 42-kD forkhead-containing highly enriched protein in thyroid follicular cells. TTF-2, encoded by the *TTF-2/foxe1* gene, is a promoter-specific transcriptional repressor [12] that displays both promoter and transcriptional activation domain specificity [22].

Forkhead domain-containing proteins are involved in embryonic pattern formation and regional specification. Forkhead genes have also been identified as factors that bind to regulatory elements in mammalian genes expressed in terminally differentiated cells [23]. Gene-targeting experiments have demonstrated that *TTF-2/foxe1* null mice exhibit cleft palate [24]. In humans, mutations of the gene encoding for TTF-2 result in Bamforth syndrome, characterized by thyroid agenesis, cleft palate, spiky hair and choanal atresia [25].

The aim of this study was to investigate the phenotype of *TTF-2* transgenic mice from E12.5 to E15.5. We demonstrated that overexpression of TTF-2 during the palatogenesis may contribute to cleft palate.

## Materials and methods

### Mouse breeding

C57BL/6J mice (Jackson Laboratory, Bar Harbor, MI, USA) were maintained at a temperature of 22°C on 12 hr light/12 hr dark cycles and were provided access to food and filtered water. C57BL/6J male and female mice (6 weeks old) were mated overnight. Vaginal plugs were observed the following morning which was considered day 0 of the embryo (E0).

### Generation of plasmid vector

Recombinant plasmid expression vector pBROAD3-*TTF-2* coding mouse TTF-2 protein was generated by our laboratory. It contained the *ROSA26* gene promoter and the 1113 bp gene fragment whose codes start from the ATG initiation codon in the 593rd nt of the *TTF-2* gene to the TGA termination codon at the 1705th nt. The plasmid pBROAD3- *TTF-2* was linked by 3,-UTR and the poly(A) signal sequence of the human EF-1α gene. The full-length of pBROAD3-*TTF-2* was 5.4 kb. The pBROAD3-*TTF-2* was transfected into the bone marrow mesenchymal cells of C57BL/6J mice. Desired protein production was verified by western blotting analysis.

### Preparing of the gene element for microinjection

The pBROAD3-*TTF-2* was digested with *Pac*I. The reaction condition was in 100 μl (400 μg) plasmid, 10× Buffer 30 μl, *Pac*I 6 μl, 0.1% BSA 30 μl, 140 μl of deionized water at 37°C or 2 hrs. Following electrophoresis with 0.8% low melting point agarose gel, the gel slice containing the 5.4 kb target gene fragment was excised to purify the DNA fragment by using the Agarose Gel DNA Extraction Kit [QIAGEN China (Shanghai) Co., Ltd., Pudong, Shanghai, China].

### Microinjection and fertilized ovum transplantation

C57BL/6J female mice (8–10 weeks old) were given intraperitoneal injection of PMSG (5 IU; Sigma-Aldrich, USA) and HCG (5 IU; Sigma-Aldrich Corp., St. Louis, MO, USA). The purified DNA was diluted in sterile PBS buffer at the final concentration of 2–6 μg Pml (about 2000 copy PPl). The dissolved DNA was microinjected into the male pronucleus of the fertilized ovum. Next, the fertilized ova were cultured and the 2-cell-stage embryos were transplanted into a corner of the uterus of the pseudopregnant C57BL/6J mice.

### Genotyping of the transgenic foetus

The genotype of the mice was determined by PCR using genomic DNA extracted from tail biopsies of the transgenic foetus. The 5′-primer sequence of *TTF-2* gene was 5′-ACTCCCAGTTCAATTACAGCTGTTG-3′. The 3′-primer sequence was 5′-GGGCGGCGGTTGGTGTTACGTTTGG-3′. The amplification was obtained with 35 cycles at 95°C for 1 min., 58°C for 1 min. and 72°C for 30 sec. after the mixture was incubated at 95°C for 10 min., followed by a final extension of 10 min. at 72°C. The PCR product was analysed by electrophoresis with 1% agarose gel (Sigma-Aldrich).

### Southern blotting identification of the transgenic foetus

The integration of the *TTF-2* transgene into the genome was determined by Southern blotting analysis using mouse-tail DNA. The recombinant plasmid expression vector pBROAD3- *TTF-2* was digested separately with *Kpn* I and *Eco*R I. This resulted in a 0.7 kb fragment as the template, and was radio-labelled for making probes. The DNA isolated from *TTF-2* transgenic mice was digested with *Eco*R I and separated by electrophoresis with 2% agarose gel (Sigma-Aldrich). The DNA was denatured, neutralized and transformed to a Hybond nylon membrane, fixed, hybridized and exposed to detect the gene by using the standard Southern blotting analysis protocol.

### RT-PCR detection of the mRNA expression of the transgenic mice

*TTF-2* mRNA was measured by quantitative real-time RT-PCR and GAPDH gene used as an endogenous control. Relative quantification of *TTF-2* mRNA was determined by using the comparative CT method (2−ΔΔCt). Data were normalized to *GAPDH* mRNA levels, and values were expressed as means ± S.E.M. (arbitrary units) of four independent groups (palatal developmental time series:E12.5-E15.5). Dissected palatal shelves were immediately frozen in liquid N_2_, disrupted in RLT buffer (Qiagen), and total RNAs were isolated by using Qiagen RNeasy Kit and Qiagen Omnisscript RT and random hexamers were used for RT reaction. Pyrobest™ DNA polymerase and the following primers were used for PCR: the 5′-primer sequence was 5′-ATTATGATTATTATTTATTATATTA′, and the 3′-primer sequence 5′-CGCGC CGCCGTCGCT GGCCGCGCCG′.

### Western blotting detection of the TTF-2 expression of the palatal tissues

Western blotting assays were performed according to the standard procedures. The palatal proteins were transferred to a PVDF membrane (Millipore Corporation, Billerica, MA, USA). The membranes were probed with primary antibody at 1:500 dilution (Goat anti-mouse TTF-2 polyclonal antibody IgG; Santa Cruz Biotechnology, Inc., Santa Cruz, CA, USA) and visualized with the secondary antibody at 1:2000 dilution (AP-Goat Anti-Rabbit IgG; Santa Cruz) and the ECL kit (Bio-Rad, Benicia, CA, USA). The membranes were stripped and re-probed with β-actin to correct for differences in protein loading. The immunoreactive protein bands were visualized on X-ray film with an enhanced chemiluminescence light (ECL)-detection system (Bio-Rad). Relative grey values of the Western blotting were quantitatively analysed by using the Bio-Rad software (Bio-Rad). Data are presented as the mean ± S.E.M. (arbitrary units) of four independent groups.

### Immunohistochemistry detection of the expression of TTF-2 of the palatal tissues

The palatal shelves of control mice and transgenic mice on E12.5 to E15.5 were fixed with 4% paraformaldehyde and embedded in paraffin. Immunohistochemistry staining was performed in accordance with the manufacturer's instructions [26] and visualized by diaminobenzidine (DAB) staining. The same primary antibody was used at 1:200 dilution. The paraffin-embedded ovary cancer using anti-TTF-2 IgG was used as a positive control. No staining was observed when omitting primary antibody or replacing the primary antibody with isotype serum as a negative control. Immunoreactivity was analysed through Image Pro Plus software (Media Cybernetics, Bethesda, MD, USA). For every section, the integral optical density of every visual field was calculated.

### Statistical analysis

Statistical analysis for all data was carried out by using the statistical package for social science (version 11.0, SPSS Inc, Chicago, IL, USA). The numerical values are presented as mean ± S.D. The results of RT-PCR, Western blotting and immunohistochemical staining were analysed with the Student's *t*-test. The comparison of means between groups was carried out by using one-way anova.

## Results

### The results of microinjection and fertilized ova transplantation

**Fig 1:**
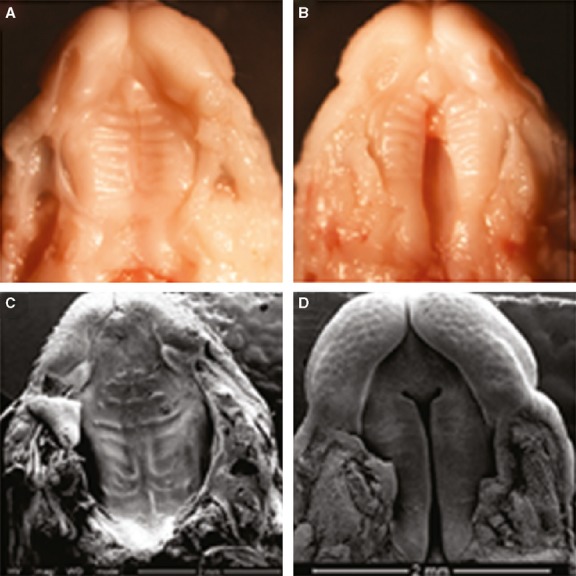
Micrographs and Scanning electron micrographs of the palatal shelves region of control mice and *TTF-2* transgenic mice. (A) and (C) showed that the palatal shelves were fused on E18 in the control groups. However, the palatal shelves were not fused on E18 in the *TTF-2* transgenic mice as shown in (B) and (D).

In this study, 1,203 fertilized ova were obtained and 982 fertilized ova were microinjected with *TTF-2* gene. A total of 580 live fertilized ova were transplanted after microinjection to the oviducts of 29 pseudopregnant female mice. In all, 68 embryos were obtained for analysis. As shown in Figure 1A and C, palatal shelves were fused on E18 in the control groups, but were not fused on E18 in the *TTF-2* transgenic mice (Fig. 1B and D).

Six founder mice were bred with C57BL/6J mice to produce offspring (F1). The F1 mice with the integrated transgene were determined by PCR and Southern blotting analysis using tail DNA as templates. F1 mice mated brother-sister produced an F2 generation for further studies. One of founder mice failed to pass the transgene to offspring, probably due to the mosaic effect on the founder mouse. In total, 42 transgenic mice with cleft palate were born and died 24 hrs after their birth.

### Gene expression determined by PCR and Southern blots

**Fig 2:**
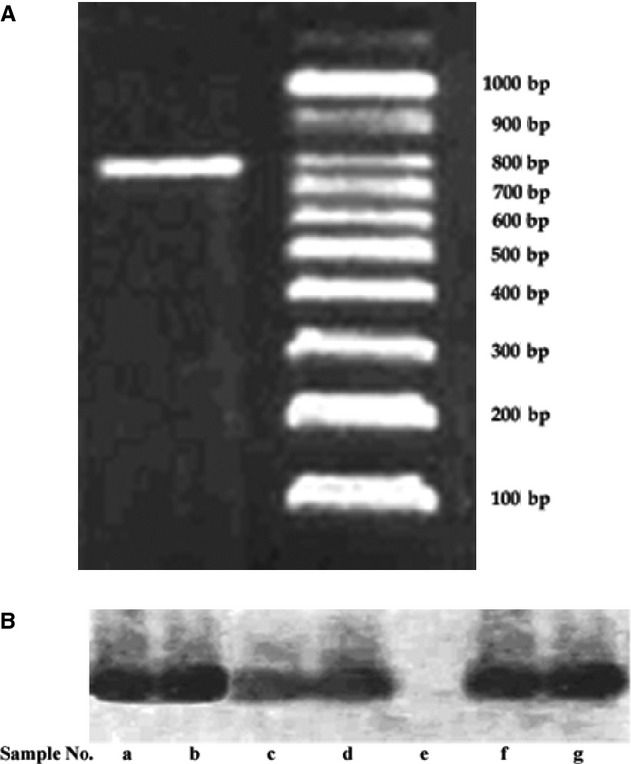
(A) Results of PCR using genomic DNA extracted from tail biopsies of the transgenic foetus. (B) Results of Southern blots detection of the genome DNA of the positive PCR transgenic mice.

Among 42 transgenic mice and 68 embryos, a 784 bp positive band was seen in all **s**amples by PCR detection (all the transgenic mice had a 784 bp positive band). Figure 2A shows the results of PCR using genomic DNA extracted from tail biopsies of the transgenic foetus. The DNA of the 72 positive *TTF-2* PCR mice was then subjected to Southern blotting analysis. Figure 2B shows the results of Southern blots of the genome DNA of the positive PCR mice.

### TTF-2 mRNA expression of the palatal tissues

**Fig 3:**
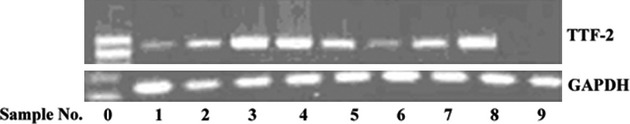
In the transgenic mice group, the 508 bp band was found on E12.5, 13.5, 14.5 and 15.5. In the control group, the 508 bp band was found on the E12.5, 13.5, 14.5 However, there was no 508 bp band found on E15.5 in the control group. 0:Marker;1: Positive control;2: E 12 Trs;3: E 13 Trs;4: E 14 Trs;5: E 15 Trs;6: E 12 Ctr;7: E 13 Ctr;8: E 14 Ctr;9: Ed15 Ctr (E: embryonic day;Trs: transgenic group;Ctr: control group).

**Fig 4:**
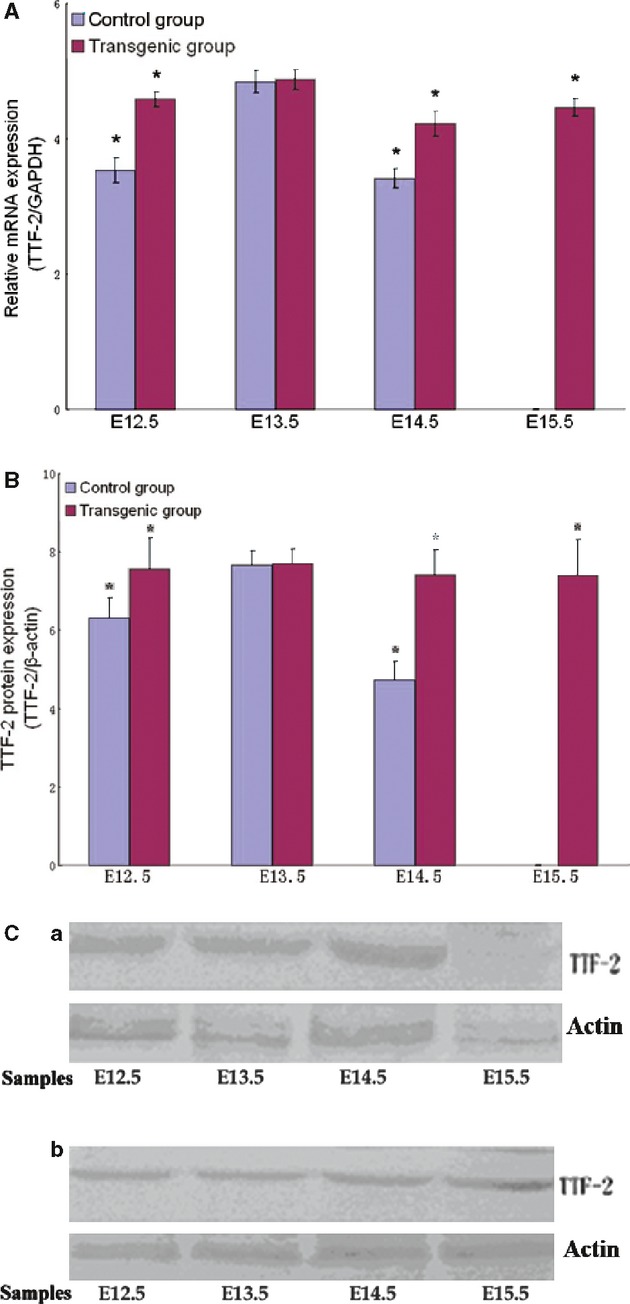
(A) Relative *TTF-2* mRNA expression in the *TTF-2* transgenic mice and the control group. As can be seen from (A), there was no obvious change on *TTF-2* mRNA expression in the *TTF-2* transgenic mice. However, there was an apparent change in the control group and reached its peak on E13.5 and started to decline later. The *TTF-2* mRNA expression was not detected on E15.5. (B) TTF-2 protein expression in the *TTF-2* transgenic mice and the control group. The change in tendency of the TTF-2 protein in the control group was basically consistent with the change in *TTF-2* mRNA level. The tendency of the TTF-2 protein expression change of the *TTF-2* transgenic mice was not obvious. (C) The Western blot results of the *TTF-2* expression of the transgenic mice and control group on E12.5, 13.5, 14.5 and 15.5.

In the transgenic mice group, the 508 bp band was found on E12.5, 13.5, 14.5 and 15.5. In the control group, the 508 bp band was found on the E12.5, 13.5, 14.5. However, there was no 508 bp band on E15.5 in the control group (Fig. 3). The results of relative *TTF-2* mRNA expression of the transgenic mice and control group mice are shown in Figure 4A. There was no statistical difference in the *TTF-2* mRNA expression changes of the transgenic mice on E12.5, 13.5, 14.5 and 15.5(*P* > 0.05). The *TTF-2* mRNA expression in the palatal tissues emerged on E12.5 in the control group. The expression level was the highest on E13.5 and began to decrease from E14.5. There was no detectable *TTF-2* mRNA expression on E15.5(*P* < 0.05). *TTF-2* mRNA expression was higher in the transgenic mice than in the control group on all other embryonic days(*P* < 0.05). As shown in Figure 4A, there was no apparent change in *TTF-2* mRNA expression in the *TTF-2* transgenic mice. However, there was a significant change in the control group mRNA expression, which reached its peak on E13.5 and started to decline thereafter. The *TTF-2* mRNA expression was not detectable from E15.5.

### Western blotting analysis

The results of TTF-2 protein expression of the transgenic mice and control group mice are presented in Figure 4B and C. The pattern of the TTF-2 protein in the control group was basically consistent with that of the mRNA level. It has been suggested that the increase in *TTF-2* transcription level is the main reason for the increased expression of TTF-2 protein. The tendency of TTF-2 protein expression of the *TTF-2* transgenic mice was not visible. Figure 4C shows the Western Blot results of *TTF-2* expression of the transgenic mice and control group on E12.5, 13.5, 14.5 and 15.5.

### Immunohistochemistry detection of TTF-2 in palatal tissues

**Fig 5:**
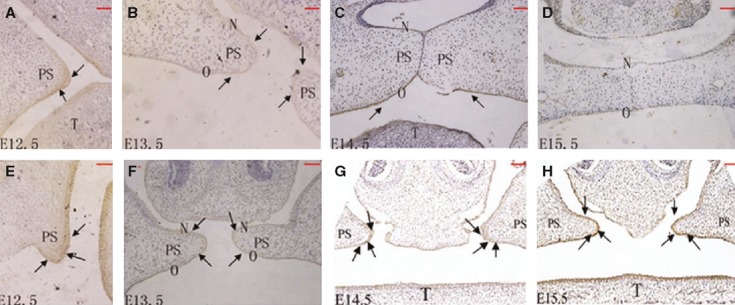
Immunohistochemical staining of TTF-2. TTF-2 expression in the palatal shelves on E12.5-15.5 in the control group mice (A–D) and in the transgenic mice (E–H).In the transgenic group, there was positive immunohistochemical staining of MEE cells, but no expression differences were found in the edge epithelium tissue of the palatine process from E 12.5 to E15.5. The positive immunohistochemical staining of MEE cells was increased in transgenic group compared with control group on the corresponding phase. N: nasal epithelium. O: oral epithelium. PS: palatal shelf. T: tongue. The positive immunohistochemical staining of MEE cells is indicated with arrows. Scale bars represent 50 μm.

The murine palatal process protruded from the maxillary process on E12.5. In the control group, there were TTF-2 positive cells confirmed by immunohistochemical staining on the epithelial cells of the palatine process. The palatine process took place on a position of verticality on E13.5. TTF-2 was expressed in the epithelial cells on the surface of the palatine process and the staining was stronger on E13.5 than that on E12.5. The palatine process took place on a horizontal position on top of the tongue on E14.5. The surface of the palatine process was a single layer of epithelial cells. However, the TTF-2 staining was weaker than that of E13.5. The palatine processes on both sides became adherent on E15.5 (Fig. 5A–D).

In the transgenic group, TTF-2 positive MEE cells remained unchanged in the palatine process from E12.5 to E15.5. The number of positive cells was increased compared with that in the control group on the corresponding phase (Fig. 5E–H).

## Discussion

It has been demonstrated that a variety of genes are involved in palatogenesis [27]. Mutations in genes are associated with regulating growth, differentiation, and EMT during the palatogenesis process, both in humans and in mouse models, all contributing to the formation of a cleft palate [28–30]. The pathogenesis of cleft palate, however, is still largely unknown, although genetic factors have been suggested.

Transgenic mice are now frequently used to create animal models to study the pathogenesis of human diseases. This system is likely to be the most sensitive assay for studying spatial and temporal regulation of a single gene product and its effect in the organism of a transgenic animal. The transgenic mouse is a particularly useful and popular model because its genome is very well characterized and can be manipulated with relative ease. However, animal models are relatively few in the field of human cleft palate. Transgenic models are valuable for studying the following points: (*i*) the identification of the tissue specificity of the expression directed by the promoter and (*ii*) the pathological effects linked to the expression of a single gene, enabling us to understand the pathogenesis of cleft palate.

The TTF-2, belonging to the forkhead/winged family, is a nucleoprotein. The pattern of TTF-2 expression during thyroid development has been extensively studied [21, 31]. Zannini *et al*. performed a detailed analysis of TTF-2 expression in thyroid precursors, to compare with the expression of TTF-1. TTF-2 is known to express in thyroid cell precursors at E 8.5 and could function as an inhibitor of differentiation in the early stages of thyroid development, perhaps by interfering with transcriptional activation of thyroid-specific gene expression by TTF-1 and, possibly, Pax-8 [21].

It was previously demonstrated that complete deletion or partial loss of its function may lead to cleft palate in both animal models (*TTF-2*^−/−^ mouse) and human beings [28–30, 32]. The *TTF-2* gene appears to play a role in normal palate development, as *in situ* expression studies [29] detect *TTF-2* transcripts in developing palatal shelves. We carried out this experiment to understand the role of TTF-2 in the formation of palate. The overexpression of *TTF-2* gene in the transgenic mice is useful for evaluating tissue specificity and temporo-spatial specificity of the *TTF-2* gene. For this reason, we established the *TTF-2* transgenic mice model for the first time, and analysed the expression patterns of the TTF-2 in the palate from E 12.5 to E 15.5.

The change in TTF-2 protein was less significant than that of mRNA, indicating that there are other regulatory mechanisms (post-transcription) involved, possibly *via* the phosphorylation of this protein. In the study, there was no significant difference in the *TTF-2* mRNA and the TTF-2 protein in the palatal shelves on E13.5 between the transgenic and the control groups(*P* > 0.05). However, on the other embryonic days, the former expressions were higher than the control ones(*P* < 0.05)and there was no temporo-spatial change.

The expressions of TTF-2 in the MEE cells during palatogenesis in the control mice were found from E12.5 to E15.5 with a temporal-spatial change. The TTF-2 mainly expressed in the epithelium nucleolus, indicating its endogenous activation. The expression peak emerged in the later period of the growth of the palatine process. In the *TTF-2* transgenic mice, it was indicated that TTF-2 had a high expression in MEE cells during the period of the palatal fusion. Previous studies revealed that the fate of the MEE, which is known to form the MES upon palatal shelf fusion, plays a very important role in palatal development. Three major mechanisms have been proposed for MEE degeneration: epithelial-mesenchymal transformation (EMT) [[6, 7, 33]]; MEE cell apoptosis (programmed cell death) [2]; and lateral migration of MEE cells [8]. Further studies will be needed to clarify how TTF-2 might affect MEE cellular functions. Does TTF-2 prevent apoptosis or epithelial to mesenchymal transformation?

The present study has demonstrated that TTF-2 was highly expressed in the palate at the key time point during palatogenesis in *TTF-2* transgenic mice. To the best of our knowledge, this is the first report of the TTF-2 expression pattern and alterations during palatogenesis in *TTF-2* transgenic mice.

The animal model established here may be useful for further studying the pathogenesis of cleft palate. In our study, we found that overexpression of TTF-2 caused the cleft palate. This might be a heredity mechanism for human cleft palate. Along with the identification of the *TTF-2* downstream target genes, we can better understand the nature of the heredity mechanism by studying the abnormal TTF-2 expression. Our study indicates that TTF-2 may be involved in the palatogenesis and pathogenesis of cleft palate.
